# The Significance of Serum CA-125 Elevation in Chinese Patients with Primary Budd-Chiari Syndrome: A Multicenter Study

**DOI:** 10.1155/2015/121060

**Published:** 2015-09-15

**Authors:** De-lei Cheng, Hao Xu, Wei-fu Lv, Rong Hua, Hongtao Du, Qing-qiao Zhang

**Affiliations:** ^1^Department of Interventional Radiology, Anhui Provincial Hospital, Hefei, Anhui 230001, China; ^2^Department of Interventional Radiology, Affiliated Hospital of Xuzhou Medical College, 99 West Huaihai Road, Xuzhou, Jiangsu 221002, China; ^3^Department of Radiology, Xuzhou Central Hospital, Xuzhou, Jiangsu 221002, China

## Abstract

*Objective*. To investigate the serum level of CA-125 and its corresponding clinical significance in Chinese patients with primary BCS. *Methods*. Serum CA-125 was measured in 243 patients with primary BCS receiving interventional treatment in the participating hospitals and in 120 healthy volunteers. The correlation between serum CA-125 levels and ascites volume, liver function, and prognosis was analyzed. *Results*. Serum CA-125 was significantly elevated in BCS patients compared to healthy volunteers (*P* < 0.001). Higher levels of CA-125 were found in BCS patients with abnormal hepatic function and low serum albumin levels and in patients with high volume of ascites compared to patients without these abnormalities. Serum CA-125 levels significantly correlated with ascites volume, serum level of alanine aminotransferase, aspartate aminotransferase, albumin, and Rotterdam BCS scores. The follow-up study indicated that the survival rate and asymptomatic survival rate after interventional treatment were lower in BCS patients with serum CA-125 > 175 U/mL (*P* < 0.05). *Conclusion*. Serum CA-125 was significantly higher in patients with primary BCS and had a positive correlation with the volume of ascites, severity of liver damage, and poor prognosis. Thus the serum CA-125 levels may be used to estimate the severity and prognosis of BCS in Chinese patients.

## 1. Introduction

Budd-Chiari syndrome (BCS) is a rare and clinically challenging disorder defined as the obstruction of hepatic venous outflow anywhere from the small hepatic veins to the suprahepatic inferior vena cava [1–3]. BCS is classified as primary or secondary depending on the cause of the obstruction. For example, primary BCS originates from an obstruction of a vein, while secondary BCS is caused by an obstruction originating outside a vein (i.e., due to compression or invasion by tumors or abscesses) [2]. BCS remains a rare disease with an incidence rate of 1-2 cases per million per year, although a study in Japan estimated a prevalence of 2.4/million with 20 new cases being diagnosed each year. The disease has higher prevalence in developing countries such as China, India, Nepal, and South Africa, with the Yellow and Huaihe river basins of China reporting the highest incidence rate of one case per 10 thousand individuals [1, 4–7]. The clinical presentation of BCS is dependent on the extent of hepatic vein occlusion, based on which the syndrome can be classified as fulminant, acute, subacute, or chronic. Prominent clinical features present in almost all patients include abdominal pain, hepatomegaly, and ascites. Nausea, vomiting, and mild jaundice are common in the fulminant and acute forms, while splenomegaly and gastrointestinal bleeding may be seen in the chronic forms. When the inferior vena cava is occluded, dilated venous collaterals are present in the flanks, along with pedal edema, varicose veins, and pigmentation of lower extremities [1, 6, 7].

Antithrombotic drugs, angioplasty, surgical stents, transjugular intrahepatic portosystemic shunt (TIPS), and liver transplantation are used for the treatment of BCS. Prognosis depends on the cause and severity of the disease. Good prognosis is observed if treated at an early stage but can be a challenge or even fatal if left untreated or when there is recurrent disease [1, 5, 6]. Therefore, accurate estimation of disease severity and prediction of prognosis are of great clinical significance.

Carcinoma antigen 125 (CA-125) is a member of mucin family glycoproteins and is used as a diagnostic and prognostic biomarker for ovarian cancer [7, 8]. The levels of serum CA-125 are elevated in certain benign conditions such as heart failure and posthepatitic cirrhosis and can be correlated to the outcome of disease [9–17]. Our previous findings indicated that the serum level of CA-125 was also elevated above the normal range (>35 U/mL) in BCS patients [18]; however, there were no further investigations on its clinical significance. It was recently reported that BCS patients with elevated serum level of CA-125 were misdiagnosed as having gynecological tumor and underwent diagnostic laparoscopy [19]. Therefore, we performed a large prospective multicenter study, which included patients with recently diagnosed primary BCS to elucidate the correlation between BCS and serum CA-125 levels from three major hospitals with highest incidence of the disease in China. It aimed to investigate the potential clinical significance of serum CA-125 in primary BCS patients.

## 2. Material and Methods

### 2.1. Patients

A total of 254 BCS patients who fulfilled the inclusion criteria were admitted in hospitals of Anhui Provincial Hospital, Affiliated Hospital of Xuzhou Medical College, or Xuzhou Center Hospital between August 2011 and April 2013. Apart from the seven cases lost to followup, four cases of secondary BCS were caused by tumors or orthotopic liver transplantation. Finally 243 patients (147 males and 96 females) with an average age of 46.3 ± 11.4 years were recruited into the study (Tables 1 and 2). Among these patients, 73 patients (42 males and 31 females) had been reported in our previous study [18]. A total of 120 healthy volunteers (67 males and 53 females) with an average age of 45.1 ± 14.2 years were randomly recruited as control. The study was conducted as per the declaration of Helsinki and was approved by ethical committees of the participating hospitals.

### 2.2. Inclusion and Exclusion Criteria

Patients were included into BCS group, if the disease was confirmed by both magnetic resonance imaging and color Doppler ultrasound diagnosis. All the patients were diagnosed as having primary BCS who had not received any medical treatment earlier. The control group had individuals with normal liver and kidney function, with no hepatic cirrhosis as indicated by Doppler ultrasound diagnosis. Individuals with history of alcoholism and poison contact, circulatory and respiratory disease, hepatitis, autoimmune hepatitis, or other chronic liver diseases, or tumor in gynecological and digestive system were excluded from the study.

### 2.3. Diagnostic Criteria

Hepatic functional abnormity was diagnosed as higher serum levels (>40 U/L) of serum alanine transaminase (ALT) and (or) aspartate aminotransferase (AST). Hypoproteinemia was diagnosed if serum albumin (ALB) was lower than the normal range (<35 g/L). Doppler ultrasound examinations and magnetic resonance imaging were carried out to diagnose the presence of ascites. The ascites was considered minimal if its presence was located under the diaphragm, around the space between liver and kidney, spleen and kidney, or bladder or rectal space. Ascites diffusely distributed among abdomen or around bowel and parenchymal organs was considered as moderate while it was considered to be in large volume if the abdominal cavity was filled with ascites [20]. Hepatocellular carcinoma was diagnosed according to pathological biopsy results or presence of a hypervascular nodule with washout during the portal venous phase of dynamic enhanced scan and higher level of serum alpha fetal protein (>400 ng/L) [21]. Rotterdam BCS scores were adopted to classify the patients in the study [22].

### 2.4. Methods

Peripheral blood samples were collected within three days after admission to the hospital and sent to the laboratory for the tests. Serum level CA-125 was determined by the method of electrochemiluminescence immunoassay (Roche, Basel, Switzerland) using Cobase411 electrochemiluminescence kit and analyzer as per the manufacturer's instructions. The serum levels of ALT, AST, ALB, bilirubin, and prothrombin time were measured using standard protocol. The volume of ascites was estimated by Doppler ultrasound. The interventional therapy such as percutaneous transluminal angioplasty or transjugular portosystemic shunt was performed within one week after admission to the hospital. All the patients were followed up every week for a month, followed by monthly follow-up examination for two months and then every month until October 2013. Loss to followup and death if any were recorded. The median followup was 15 months (range, 6–24).

### 2.5. Statistical Analysis

Quantitative data was presented as mean ± SD. Independent sample *t*-test was carried out for quantitative comparison between the two groups, one-way ANOVA was used for the comparison among multiple groups if the normality was confirmed by Kolmogorov-Smirnov test or else, Wilcoxon W rank test was used for two-group comparison, and Kruskal-Wallis test was used for comparison among multiple groups. Friedman test was utilized for quantitative data comparison from a number of related samples. Pearson test and Spearman test were utilized for correlation analysis between two groups of quantitative data or between quantitative data and hierarchical data, respectively. The survival rate of patients was calculated by Kaplan-Meier method, and confidence interval (CI) was calculated by exact probability computation and normal approximation method, whilst the survival rate comparison between two groups was analyzed by Log-Rank *χ* ^2^ test. *P* < 0.05 was considered as statistical significance. All statistical analyses were carried out in SPSS for Windows version 16.0 (SPSS Inc., Chicago, IL, USA).

## 3. Results

### 3.1. Baseline Characteristics

The clinical features and risk factors of the BCS patients are detailed in Tables 1 and 2. BCS patients (*N* = 243 including 147 males and 96 females) with an average age of 46.3 ± 11.4 years and 120 healthy volunteers (67 males and 53 females) with an average age of 45.1 ± 14.2 years were recruited into the study. There was no statistical significance between two groups in terms of age (*χ* ^2^ = 0.828, *P* = 0.363) and gender (*t* = 0.338, *P* = 0.812).

### 3.2. Serum CA-125 Levels in BCS Patients and Healthy Volunteers

**Figure 1:**
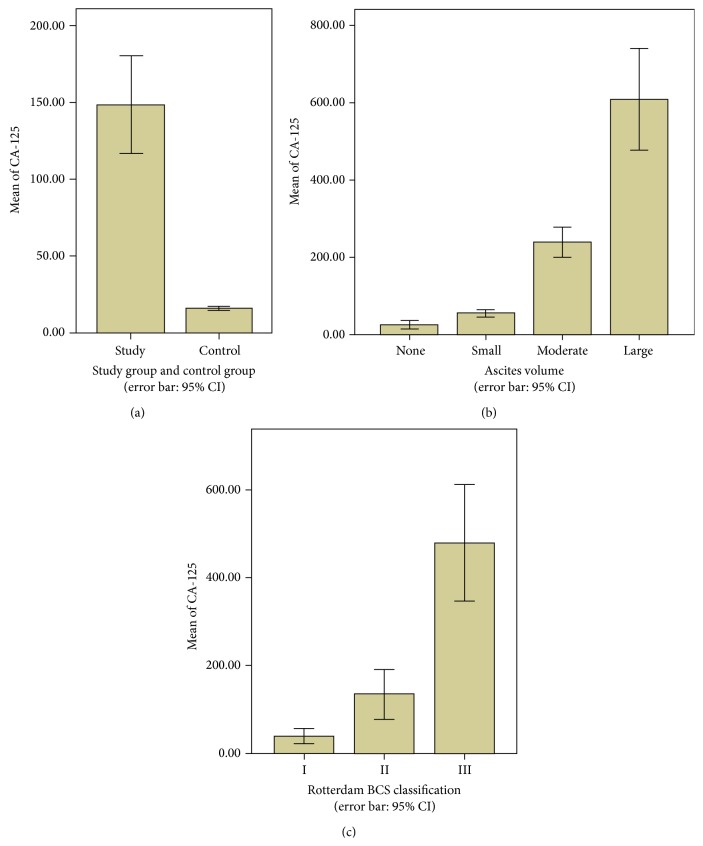
Serum level of CA-125 (U/mL). (a) The comparison between patients with Budd-Chiari syndrome (BCS) (*n* = 243) and healthy volunteers (*n* = 120). (b) The comparison among BCS patients with different volume of ascites, no ascites (*n* = 108), small volume (*n* = 75), moderate volume (*n* = 21), and large volume (*n* = 39). (c) The comparison among BCS patients with different Rotterdam BCS score, grade I (*n* = 107), grade II (*n* = 99), and grade III (*n* = 37).

The average serum CA-125 was 147.9 ± 246.6 U/mL in BCS patients, which was significantly higher than that in healthy volunteers, 16.0 ± 7.2 U/mL (*Z* = 10.1, *P* < 0.001) (Figure 1(a)).

### 3.3. Difference of Serum CA-125 among BCS Patients

Serum CA-125 levels were significantly higher in patients with abnormal hepatic function (*N* = 65, 307.7 ± 444.7 U/mL) and low ALB levels (*N* = 66, 285.0 ± 303.0 U/mL) and those with ascites (*N* = 135, 242.7 ± 300.1 U/mL) compared to corresponding patients who had normal hepatic function (86.9 ± 174.2 U/mL) and normal serum ALB (97.7 ± 201.1 U/mL) or without ascites (20.7 ± 12.3 U/mL) (*P* < 0.05). Concurrent hepatic carcinoma with BCS was found in 15 patients; however, their serum CA-125 was not different compared to the other 228 BCS patients (*Z* = 0.801, *P* = 0.423) (Table 3).

### 3.4. Correlation between Serum CA-125 Levels and Severity of BCS Patients

BCS patients were classified into four groups based on the volume of ascites at the time of admission. The serum CA-125 levels were 20.7 ± 12.3 U/mL in patients without ascites (*N* = 108), 68.15 ± 71.6 U/mL (*N* = 75) in patients with small volume of ascites, 251.4 ± 89.8 U/mL (*N* = 21) in patients with moderate ascites, and 573.5 ± 360.1 U/mL (*N* = 39) in patients with large volume of ascites. Significant difference was observed among four groups (*χ* ^2^ = 153.54, *P* < 0.001) (Figure 1(b)).

Patients were classified into three groups in terms of Rotterdam BCS scores at admission. Grade I BCS patients (*N* = 107) had a serum CA-125 level of 39.4 ± 81.5 U/mL, while the levels in grade II patients (*N* = 99) were 134.5 ± 299.4 U/mL and that in grade III patients (*N* = 37) was 478.6 ± 374.6 U/mL. The difference between the three groups was observed to be statistically significant (*χ* ^2^ = 119.818, *P* < 0.001) (Figure 1(c)).

A positive correlation was observed between serum CA-125 and ascites volume, serum AST, ALT, and Rotterdam BCS scores, with a correlation coefficient of 0.79, 0.45, 0.29, and 0.71, respectively (*P* < 0.001), whereas the negative correlation was seen between serum CA-125 and ALB levels, with correlation coefficient of −0.393 (*P* < 0.001).

### 3.5. Prognostic Difference among BCS Patients after Intervention Therapy

**Figure 2:**
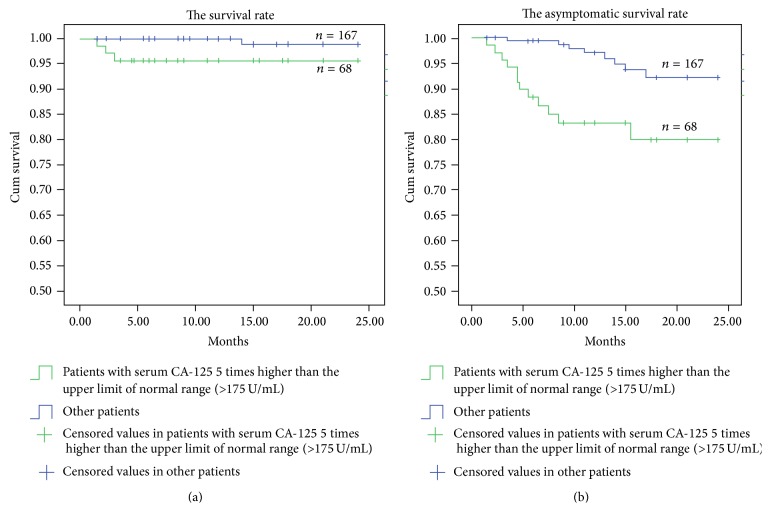
Survival rate curve after interventional treatment of Budd-Chiari syndrome patients with different serum CA-125.

For eight patients, interventional treatment failed due to inaccessibility of the target vein for long-segment occlusion. Therefore they were only treated with medicine (i.e., diuretic drugs, hydrochlorothiazide). The other 235 (96.7%) patients were treated with interventional methods (angioplasty in 223 cases; TIPS in 12 cases), and the technical success rate was 96.7%. Of these 235 patients, serum CA-125 was five times higher than the upper limit of normal range (>175 U/mL) in 68 patients, who were classified as high CA-125 group while the other 167 patients were classified into lower CA-125 group. The survival rate and asymptomatic survival rate after interventional treatment were significantly lower (*P* < 0.05) for patients in high CA-125 group (95.6% (95% CI: 90.7%–98.9%) and 79.8% (95% CI: 69.5%–90.1%)), in comparison with the lower CA-125 group (98.8% (95% CI: 96.4%–99.9%) and 92.0% (95% CI: 86.5%–97.5%)) (Table 4, Figures 2(a) and 2(b)).

### 3.6. Serum CA-125 before and after Intervention Therapy

BCS associated symptoms were alleviated in all the patients who were treated with interventional methods, with the exception of 3 patients who did not show improvement. Complete remission was observed in 219 patients, while 13 patients showed marked improvement after intervention therapy. The pre- and postoperative level of serum CA-125 in 3 BCS patients who had no response to interventional treatment had no significant difference (842 U/mL versus 937 U/mL, 981 U/mL versus 855 U/mL, and 764 U/mL versus 660 U/mL, resp.). Among these patients, two patients died in two months after interventional treatment, and the other patient survived to the last followup with a moderate amount of ascites. The average level of serum CA-125 in these 232 patients who had response to intervention therapy reduced to 39.8 ± 48.6 U/mL at the time of discharge from hospital, significantly lower than the levels (141.4 ± 222.8 U/mL) at admission (*Z* = −9.721, *P* < 0.001). During the followup 16 patients had recurrent symptoms and 4 patients died. The average serum level of CA-125 in these patients was 451.2 ± 492.5 U/mL, 70.4 ± 83.1 U/mL, and 371.0 ± 369.9 U/mL, at the time of admission, discharge, and readmission, respectively. The difference in levels of CA-125 was statistically significant (*χ* ^2^ = 25.0, *P* < 0.001).

## 4. Discussion

In this study, we report elevated levels of CA-125 antigen in serum of BCS compared to healthy controls. Similar observations have been reported in other hepatic diseases in earlier studies [13–17]. Further our results suggest the correlation between serum CA-125 levels and extent of liver damage, as indicated by the volume of ascites, serum ALT, ASL, and ALB levels. We also observed a poor outcome in BCS patients with higher serum CA-125 levels.

Hepatic injury is often found in BCS patients because of obstruction of hepatic venous outflow. Acute obstruction could result in congestion, edema, and necrosis of hepatocytes and lead to liver cirrhosis [20]. Serum levels of ALT and AST are the biomarkers of liver damage, which are released from hepatocytes into serum when liver gets damaged, while the biosynthesis of ALB in hepatocytes is decreased under similar conditions. Our study suggests that BCS patients who had abnormal liver function or decreased ALB had elevated serum CA-125 as well. The correlation analysis confirmed that there was positive correlation between CA-125 and AST or ALT and negative correlation between CA-125 and ALB, implying that serum CA-125 of BCS patients was correlated to the severity of liver damage. Our findings were also consistent with what had been found in HBV induced cirrhosis [13, 17].

The use of serum CA-125 levels as a biomarker to monitor liver carcinogenesis from cirrhosis is still under deliberation [17]. In the current study, there were 14 BCS patients complicated with hepatocellular carcinoma, whose serum CA-125 was not significantly different compared to those without concurrent hepatocellular carcinoma. Our findings were consistent with other studies which found no correlation between serum CA-125 and liver carcinogenesis in BCS patients, although the number of patients was low in the current study [17].

CA-125 is highly glycosylated protein produced by epithelial cells. Elevated serum CA-125 is reported to be associated with ascites formation in patients of liver cirrhosis [13–17]. We also observed a significant association between volume of ascites and serum level of CA-125 in BCS patients. Therefore, we thought the elevated level of serum CA-125 in BCS was also associated with ascites. However, the mechanism of increased CA-125 and ascites formation is not elucidated yet. It is assumed that mesothelial cells of peritoneum, pleura, and pericardium could synthesize and release CA-125 besides ovarian epithelial cells. Peritoneal mesothelial cells under the pressure of ascites overexpress CA-125 and eventually release the antigen into peripheral blood resulting in elevated level of serum CA-125 [13–15]. Supporting evidence for this hypothesis comes from the fact that serum CA-125 levels rapidly decrease within 48 hours of ascites releasing therapy and the level of ascites CA-125 is positively correlated with the volume of ascites [13–15].

Liver function and ascites formation have been related to prognosis of BCS patients [22, 23]. Elevated serum ALT levels, volume of ascites, hepatic encephalopathy, total bilirubin level, and prothrombin time could be associated with poor outcome in BCS patients. The Rotterdam score was created for BCS prognosis using a multivariate analysis. Ascites and hepatic encephalopathy were scored as present or absent and prothrombin time as higher or lower than an INR of 2.3. Patients were divided into three classes: class 1 (good prognosis), class 2 (intermediate prognosis), and class 3 (poor prognosis) based on the score.

In the current study, we observed a positive correlation between Rotterdam BCS score and serum CA-125 at admission, with the correlation coefficient of 0.71. There were 68 patients with serum CA-125 higher than 175 U/mL (5 times higher than upper limit of normal range), and their survival rate and asymptotic survival rate after interventional therapy were lower than the other patients, suggesting that the elevation of CA-125 was associated with an unfavorable outcome. Serum CA-125 was also found to correlate with ascites volume and severity of liver damage, the latter two being risk factors for poor outcome of BCS. Therefore, elevated serum CA-125 could be one indicator of poor prognosis.

The BCS symptoms were greatly alleviated after intervention therapy in most of patients, and serum CA-125 levels decreased significantly when the patients were discharged from hospital. Recurrence of symptoms was associated with elevated levels of CA-125 further confirming the importance of this antigen in disease severity. Similar observation was also reported by Leggio et al. [24] in a cirrhosis patient, with increased pressure of portal vein, serum CA-125 levels decreased when portal hypertension was alleviated. Therefore, the level of serum CA-125 in BCS patients should be detected at admission, discharge, and followup. Before interventional therapy, the serum of CA-125 may be used to estimate the severity of BCS and the interventional treatment outcome; at discharge, it could be used to estimate the therapeutic effect of interventional therapy; during the followup, it could be regarded as one indicator to observe the recurrence of BCS.

There were also some limitations in the current study. These observations were carried out in Chinese patients with BCS. Considering the difference between Chinese and Caucasian patients in terms of clinical feature and etiology of BCS, our findings might not be suitable to interpret the clinical data of patients from Western countries. The research period of current study was long, and the followup was relatively shorter for some patients, which might have some effects on survival rate calculation. However, recurrence and mortality due to BCS generally occur within a year of intervention, suggesting that prolonged followup may not have additional advantages [1, 25–27]. Current study was conducted at multiple centers and serum CA-125 was measured by different technicians. However, the same types of equipment and reagents were used across hospitals, and all technicians were trained and operated strictly according to the instruction to minimize manual variations in the assay.

In summary, serum CA-125 was significantly higher in Chinese patients with primary BCS and had a positive correlation with the volume of ascites, severity of liver damage, and Rotterdam BCS prognostic rating score. The elevated serum CA-125 was associated with poor prognosis of the patients. Thus the serum CA-125 levels may be used to estimate the severity of BCS and the interventional treatment outcome, and meanwhile it could be regarded as one indicator to observe the recurrence of BCS.

## Figures and Tables

**Table 1:** Clinical data of 243 patients with Budd-Chiari syndrome at diagnosis.

	*N* (%)		Mean ± SD
Acute/chronic^*^	31/212 (12.8%/87.2%)	Duration of symptoms before diagnosis (months)	112.2 ± 109.1
Abdominal pain	52 (21.4%)	Age (years)	46.3 ± 11.4
Distension of abdomen	73 (30.0%)	Aspartate aminotransferase (U/L)	41.4 ± 33.6
Gastrointestinal bleeding	42 (17.3%)	Alanine aminotransferase (U/L)	33.5 ± 26.4
Anorexia	42 (17.3%)	Albumin (g/L)	37.1 ± 7.6
Leg edema	131 (53.9%)	Alkaline phosphatase (U/L)	136.5 ± 146.4
Leg pigmentation	108 (44.4%)	Total bilirubin (*μ*mol/L)	48.5 ± 86.4
Leg ulcer	32 (13.2%)	Direct bilirubin (*μ*mol/L)	21.6 ± 46.9
Abdominal wall distended veins	125 (51.4%)	Glutamyl peptide transferase (U/L)	117.6 ± 109.7
Leg varices	105 (43.2%)	Prothrombin time (s)	14.7 ± 7.2
Hepatomegaly	67 (27.6%)	White blood cell (×10^9^/L)	4.6 ± 3.4
Location of outflow obstruction		Hemoglobin (g/L)	117.4 ± 26.3
IVC^a^	16 (6.6%)	Platelet (×10^12^/L)	141.3 ± 97.6
HV^b^	59 (24.3%)	Alpha fetal protein (ng/mL)	17.3 ± 74.4
Both	168 (69.1%)	Spleen diameter (cm)	15.3 ± 3.2

^*∗*^Acute group: duration of symptoms ≤6 months; chronic group: duration of symptoms >6 months.

^a^HV = hepatic veins; ^b^IVC = inferior vena cava.

**Table 2:** Risk factors present in 243 patients with Budd-Chiari syndrome (patients could have more than one factor registered).

Risk factors	*n*/*n*	%
Thrombophilia		
Myeloproliferative disorder^*^	9/167	5.1
Polycythaemia vera rubra	4/167	2.3
Essential thrombocythemia	3/167	1.7
JAK2 mutation	9/167	5.1
Factor V Leiden mutation	0/167	0.0
Prothrombin G20210A mutation	0/167	0.0
Paroxysmal nocturnal hemoglobinuria	1/167	0.6
Protein C deficiency^#^	1/120	0.8
Protein S deficiency^#^	0/120	0.0
Antithrombin deficiency^#^	0/120	0.0
Antiphospholipid antibodies	43/243	17.7
Hyperhomocysteinemia^##^	51/243	21.0
Systemic		
Systemic lupus erythematosus	2/243	0.8
Ulcerative colitis	1/243	0.4
Phlebitis	3/243	1.2
Ankylosing spondylitis	1/243	0.4
Hormonal factors (women only)		
Oral contraceptive use	1/96	1.0
Pregnancy within 3 months before diagnosis	3/96	3.1
MO^###^	141/243	58.0
MOVC^a^	16/243	6.6
MOHV^b^	27/243	11.1
MOVC and hepatic vein involved	98/243	40.3
Idiopathic	47/243	19.3

^*^9 cases of JAK2 mutation, including four cases of polycythemia vera and three cases of essential thrombocythemia.

^#^Deficiency was diagnosed as nonacquired only if 1 protein was deficient, the result occurred in the absence of anticoagulants or oral contraceptive use, and the patient did not have liver dysfunction (bilirubin level 2 times the upper limit of normal).

^##^When the blood homocysteine concentrations were higher than 15 umol/L.

^###^MO = membranous obstruction.

^a^MOVC = membranous obstruction of the inferior vena cava.

^b^MOVH = membranous obstruction of the hepatic venous.

**Table 3:** Serum CA-125 level differed in patients with Budd-Chiari syndrome (mean ± SD).

	Ascites		Liver function^*^	
	Yes (*n* = 135)	No (*n* = 108)	Normal (*n* = 178)	Abnormal (*n* = 65)
CA-125 (U/mL)	242.7 ± 300.1	20.7 ± 12.3	86.9 ± 174.2	307.69 ± 444.7
*Z* ^#^	10.4		−4.14	
*P*	0.000		0.000	

	Complicated with hepatocellular carcinoma^##^		Albumin^**^	
	Yes (*n* = 15)	No (*n* = 228)	Normal (*n* = 177)	Abnormal (*n* = 66)

CA-125 (U/mL)	184.4 ± 423.3	145.2 ± 229.9	97.7 ± 201.1	285.0 ± 303.0
*Z* ^#^	0.80		−6.08	
*P*	0.423		0.000	

^#^Data in the table not well represented by a normal distribution by Kolmogorov-Smirnov test and tested by Wilcoxon W rank test.

^*^Serum alanine transaminase and (or) aspartate aminotransferase higher than upper limit of normal range (>40 U/L), regarded as abnormal function of liver.

^**^Serum albumin lower than lower limit of normal range (<35 g/L), regarded as decreased serum albumin.

^##^Budd-Chiari syndrome complicated with hepatocellular carcinoma: indicated by pathological biopsy results or a nodule with typical imaging features (hypervascular nodule with washout during the portal venous phase of dynamic enhanced scan) and a serum alpha fetal protein level greater than 400 ng/L.

**Table 4:** Survival rate comparison between Budd-Chiari syndrome patients with different serum CA-125 after interventional treatment.

	Increased CA-125 group^*^		Another group^*^		*χ* ^2^	*P*
	(>175 U/mL, *n* = 68)		(≤175 U/mL, *n* = 167)			
	Rate	95% confidence interval	Rate	95% confidence interval		
Survival rate						
3 months	95.6%	90.7%–98.9%	100%	97.4%–100%	4.33	0.037
6 months	95.6%	90.7%–98.9%	100%	97.4%–100%		
12 months	95.6%	90.7%–98.9%	100%	97.4%–100%		
24 months	95.6%	90.7%–98.9%	98.8%	96.4%–99.9%		
Asymptotic survival rate						
3 months	95.6%	90.8%–99.4%	100%	97.4%–100%	10.63	0.001
6 months	88.2%	80.5%–95.9%	99.4%	96.8%–99.9%		
12 months	83.1%	74.3%–91.9%	97.0%	94.1%–99.8%		
24 months	79.8%	69.5%–90.1%	92.0%	86.5%–97.5%		

^*^The Budd-Chiari syndrome patients with serum CA-125 5 times higher than the upper limit of normal range (>175 U/mL) were classified into group of increased CA-125; the other patients were classified into another group.

## Abbreviations

ALB: Albumin
ALT: Alanine transaminase
AST: Aspartate aminotransferase
BCS: Budd-Chiari syndrome
CA-125: Carcinoma antigen 125
CI: Confidence interval
TIPS: Transjugular intrahepatic portosystemic shunt.
